# Improving Temperature Adaptation for Food Safety: Colorimetric Nanoparticle-Based Time–Temperature Indicators (TTIs) to Detect Cumulative Temperature Disturbances

**DOI:** 10.3390/foods14050742

**Published:** 2025-02-22

**Authors:** Gustavo Lanza, Jaime Andres Perez-Taborda, Alba Avila

**Affiliations:** 1 Centro de Microelectrónica (CMUA), Departamento de Ingeniería Eléctrica y Electrónica, Universidad de los Andes, Bogotá 111711, Colombia; galanza@ucundinamarca.edu.co; 2 Área de Ciencias Básicas, Facultad de Ingeniería, Universidad de Cundinamarca, Fusagasugá 252211, Colombia; 3 Grupo de Nanoestructuras y Física Aplicada (NANOUPAR), Universidad Nacional de Colombia Sede De La Paz, La Paz 202010, Colombia; jperezta@unal.edu.co

**Keywords:** nanodispersion, colorimetric response, TTI, cold chain monitoring

## Abstract

The global commitment to ending hunger by 2030 has driven Colombia to align its Sustainable Development Goals (SDGs) toward reducing food waste and ensuring access to safe, nutritious food. A critical need is monitoring cumulative temperatures across food supply networks, prioritizing products over transport or storage infrastructure. This study introduces a Functional Time–Temperature Indicator (TTI) using nanodispersions of silver (Ag) and gold (Au) nanoparticles housed in 3D-printed plant-based resin containers. Nanoparticles were synthesized via three methods: in situ reduction (AgNPs), seed-based thermal synthesis (AgTNPs), and pulsed laser ablation in liquid (AuNPs). The TTIs operate through three colorimetric mechanisms: NP concentration, geometry changes, and agglomeration. At 4 °C, AgNPs and AgTNPs maintained stable color, while at 22 °C, they exhibited significant changes, with AgNPs reaching 252% variation within 5 h. AuNPs responded at lower temperatures, showing up to 27% variation. Containers enabled effective nanodispersion incorporation due to their thermal and optical properties. AgTNP-based TTIs demonstrated the most noticeable changes at 22 °C, with a total color difference (ΔE) of 39.9, easily detectable by observers. These TTIs provide robust solutions for continuous cold chain monitoring, enhancing food safety and preserving quality throughout the supply chain.

## 1. Introduction

Recently, the Food and Agriculture Organization of the United Nations (FAO) reported that approximately 13% of the world’s food production is lost between harvest and retail outlets, with an additional 17% wasted in households, food service establishments, and retail settings [1]. This issue is notably pronounced in Colombia, where an estimated 6.1 million tons of food incur annual losses [2,3], primarily linked to factors affecting quality. Factors contributing to food loss and waste in Colombia include inadequate infrastructure, transportation inefficiencies, and suboptimal storage facilities [4]. This situation presents a significant opportunity to engage public and private stakeholders in implementing innovative strategies to mitigate food loss and waste. It is critical to take swift action to promote more resilient and sustainable food systems, particularly given that only five years remain to achieve the Sustainable Development Goals (SDGs) established by the United Nations [5]. The SDGs are a global framework aimed at addressing key challenges such as poverty (SDG 1), hunger (SDG 2), and good health and well-being (SDG 3), among others. This underscores the urgency of accelerating efforts to tackle these pressing global issues and ensure a more sustainable future.

Insights gleaned from food producers shed light on a significant factor contributing to the decline in food quality: inadequate temperature control and monitoring across the supply chain [6]. Consequently, the importance of precise temperature regulation within designated cold chains emerges as a pivotal requirement for effective food preservation [7]. Addressing the challenge of ensuring cold chain integrity and minimizing food losses necessitates the establishment of comprehensive temperature traceability throughout the entire produce management process. A cold chain is delineated as an uninterrupted, temperature-controlled supply-chain process tailored explicitly for the preservation and transportation of perishable goods [8]. By effectively preventing processes of biological decomposition and degradation [9,10], cold chains play a crucial role in maintaining the quality and safety of perishable items.

Temperature monitoring in cold chains can be achieved through various methods. For instance, conventional probe thermometers, temperature labels, and chemical sensors are commonly employed [11]. However, these technologies typically do not allow for cumulative temperature measurement, consequently impacting the traceability of the product’s exposure temperature. A development in contact thermal sensing is the utilization of external contact sensors known as Time–Temperature Indicators (TTIs). These TTIs serve as external devices that can be easily attached to packaging, playing a crucial role in indicating changes in perishable products [12]. They provide information about remaining shelf life by monitoring and recording the effects of temperature accumulation. The duration from activation to termination of TTIs is typically indicated by an irreversible change in a physical characteristic, such as a visible color change, directly correlated with the temperature and time history [13,14]. TTIs provide a cost-effective solution, as they are affixed to the packaging of each food item. Their functionality allows for easy reading by users of any type, whether they be consumers or producers.

To date, a variety of metal nanoparticle (MNP)-based sensing solutions have been developed with varying degrees of success [15,16,17]. Specifically, in the fabrication of TTIs, the potential of metal NPs have demonstrated unique optical features when dispersed in solutions determined mainly by their Localized Surface Plasmon Resonances (LSPRs) [18]. In a system of suspended NPs (nanodispersion), LSPRs and, consequently, the optical response of the system can be externally modified by temperature in two ways: through collective variations of the system (distance between NPs) and individual variations of the NPs (morphology) [19,20]. Changes in distance are related to agglomeration or aggregation processes in the nanodispersion [21]. Morphological changes refer to shape alterations that the NPs can undergo [22]. Temperature induces variations in the nanodispersion, which can be macroscopically detected by observing a color change.

Studies have explored TTIs employing nanodispersions to monitor changes over time and temperature. Zeng et al. [22] synthesized Ag triangular nanoplates with an average thickness of approximately 5 nm and edge lengths of between 30 and 60 nm. These NPs were utilized in TTIs, with temperature-induced changes causing a blue shift in dipole resonance peak position. Exposure to 80 °C for varying durations (0 h, 1 h, 4 h, and 9 h) transformed the sharp corners of Ag nanoplates into circular disks, resulting in an LSPR peak shift of up to 320 nm at different temperatures (4 °C, 25 °C, and 47 °C). Similarly, Lim et al. [23] produced TTIs using AuNPs in gelatin, with the gelatin concentration (1% to 6%) and pH (3, 5, and 9) affecting the color signal at 30 °C for different time periods. The LSPR peak shifted from 535 to 552 nm at 1% gelatin. Wang et al. [24] developed TTIs for −20 °C storage for up to 90 days, where the inclusion of AuNPs in gelatin influenced nanoparticle size (temperature-dependent) and shape (time-dependent). Other studies have utilized various matrices for AuNP-based TTIs. Wang et al. [25] employed alginate to produce hydrogel matrices, resulting in LSPR peak shifts from 555 to 569 nm at various temperatures (−20 °C, 0 °C, and 40 °C). Additionally, Zhang et al. [26] used Ag-coated Au nanorods for TTIs with tunable color changes at room temperature by adjusting reaction parameters, including Au nanorod concentrations, cetyltrimethylammonium chloride (CTAC), ascorbic acid (AA), and pH. This study aimed to develop a plasmonic time–temperature indicator (TTI) using the chronochromic behavior of Ag shell heteroepitaxy on Au nanorods, enabling a red-to-green color change to monitor perishable deterioration and ensure reliable shelf-life tracking.

In the literature [22,23,24,25,26], nanodispersions have been identified as a type of TTI owing to their opto-thermal responses. However, we propose that for a TTI to be deemed functional, the nanodispersion must be enclosed within a container possessing specific characteristics, such as transparency and thermal conduction. Such a container facilitates the effective attachment of the nanodispersion to product packaging. In this context, we introduce a functional TTI system comprising both the nanodispersion and a container, designed to exhibit compatibility in their opto-thermal properties. This compatibility ensures precise application in colorimetric sensing.

In this study, we developed functional TTIs using nanodispersions of silver—specifically, AgNPs (silver nanoparticles) and AgTNPs (silver triangular nanoparticles)—as well as nanodispersions of gold AuNPs (gold nanoparticles) as the active materials, integrated with a container. Two types of NPs and three synthesis methods were employed, and the nanodispersions were subsequently packed into containers manufactured using 3D printing technology. By utilizing plant-based resin in the manufacturing process and employing stereolithography techniques, containers with optimal optical, thermal, and permeability characteristics were produced to encapsulate the nanodispersions and showcase their optical response. To assess the optical response of these functional TTIs, experiments were conducted at temperatures of 4 °C and 22 °C (room temperature) over 5 h to simulate disruptions in the cold chain. The time and temperature values were chosen because many fruits cultivated in Colombia, such as peaches, feijoas, strawberries, apples, blackberries, pears, and cape gooseberries, require storage at or below 4 °C, with any deviation potentially causing spoilage within hours [27]. Colombia’s diverse climate is ideal for fruit cultivation, but its fluctuating ambient temperatures, reaching up to 22 °C, present a significant challenge to fruit preservation [28]. To address this, a well-regulated cold chain is essential for maintaining proper storage and transport conditions, minimizing spoilage, extending shelf life, and preserving quality. The experimentation identified three strategies for the nanodispersions: variations in concentrations, shapes, and agglomeration processes. TTIs based on nanodispersions demonstrated colorimetric responses correlated with exposure to time and temperature through these approaches. The proposed TTI can be easily integrated into the cold chain system. It visually indicates any temperature inconsistencies by changing color when exposed to deviations beyond the specified time and temperature range. This real-time detection provides immediate feedback to users, helping prevent spoilage and ensuring that fruits are maintained under optimal conditions throughout the supply chain.

## 2. Materials and Methods

### 2.1. NP Synthesis

Nanoparticles of Ag and Au were used for their plasmonic response (LSPR), falling within the visible spectrum. Both chemical and physical methods were chosen for their synthesis to enable a comparative analysis of different routes. Specifically, AgNPs were chemically synthesized using two distinct methods: the in situ reduction method and the seed-based thermal synthetic method (NPs synthesized by this method are termed AgTNPs for distinction purposes). Additionally, AuNPs were synthesized via a physical approach known as the Pulsed Laser Ablation in Liquid (PLAL) technique.

For the in situ reduction method [29], two initial solutions were prepared. The first solution involved dissolving 17 mg of AgNO_3_ in 10 mL of H_2_O to obtain a concentration of 0.01 M, which was further diluted to 0.001 M. The second solution was prepared by dissolving 7.6 mg of NaBH_4_ in 10 mL of H_2_O to obtain a concentration of 0.02 M, which was then diluted to 0.002 M. The synthesis process involved adding 10 mL of the 0.001 M NaBH_4_ solution to a pre-cooled Erlenmeyer flaskon ice and allowing it to equilibrate for 20 min. Then, 3 mL of AgNO_3_ was slowly added at a rate of approximately 1 drop per second for around 2 min.

The seed-based thermal synthetic method involved the reduction of silver nitrate (AgNO_3_) by L-ascorbic acid in the presence of Ag seeds, polyvinyl pyrrolidone (PVP), and sodium citrate [30,31]. In the preparation of Ag seeds, 176 mL of an aqueous solution containing 0.11 mM AgNO_3_ and 2.05 mM trisodium citrate were used. Under magnetic stirring, an aqueous solution of NaBH_4_ (4.8 mL, 5 mm) was added all at once. The stirring was stopped after 10 min, and the seeds were used after aging for 5 h. For the synthesis of nanoparticles, 100 mL of Milli-Q water was stirred magnetically while sequentially adding the following solutions: 2.5 mL of 5 mM AgNO_3_, 7.5 mL of 0.8 mM vinyl pyrrolidone, 7.5 mL of 30 mM sodium citrate, and 0.2 mL of Ag seed solution. Finally, 62.5 mL of 1 nM L-ascorbic acid was added dropwise. Seed solution was prepared with silver nitrate and trisodium citrate in 11 mL of water at 0.1 mM and 2 mM, respectively. Next, under magnetic stirring, 0.3 mL of a solution of sodium borohydride (NaBH_4_) in water at 5 mM was added all at once.

AuNPs were synthesized via the pulsed laser ablation in liquid (PLAL) technique. This method involved the utilization of a high-power laser that was precisely directed toward a metallic gold target positioned at the bottom of a beaker filled with liquid [32]. By irradiating the gold target with the laser, the liquid medium was heated, leading to a reaction where tiny fragments of the target material were released as nanoparticles. For this process, an Nd-YAG (Neodymium-doped Yttrium Aluminum Gamet) laser operating at 532 nm, a pulse duration of 9 ns, and a frequency of 10 Hz was employed. The experimental details of how these samples were obtained are available in [33]. The laser beam was directed towards a mirror, reflected, then passed through a lens placed inside a beaker containing ultrapure type I Milli-q water. A 5N gold target was positioned at the base of the beaker, resting on a motorized turntable. Parameters were maintained constant throughout the synthesis, including the volume of water (15 mL), the laser pulse ablation energy (75 mJ), the ablation time (10 min), the distance between the mirror and the objective lens, and the size of the incident spot on the objective lens.

### 2.2. Container Manufacturing

Containers used to store the synthesized nanodispersions were produced by UV laser-based 3D printing. This technique employs a liquid resin that undergoes curing to craft complex 3D structures. This approach involves cutting a 3D model into individual layers, which are then sent to the SLA (stereolithography) machine [34]. The containers were 3D-printed using plant-based resin, featuring walls with a thickness of 1 nm, a cubic geometry (dimensions of 2.1 cm × 1.1 cm × 0.5 cm), and an internal volume of 1 mL. During the container printing process, a hole with a precise 0.6 mm diameter was incorporated on one side. This aperture allowed for the injection of 1 mL of the nanodispersions using a syringe fitted with a 25-gauge needle, which is commonly used for fine and precise injections.

### 2.3. Characterization Techniques

The optical responses of the nanodispersions over time at the study temperatures were examined using UV-Visible Absorbance Spectroscopy (UV-Vis) with a Thermo Scientific 10S UV-Vis Spectrophotometer (Thermo Scientific, Waltham, MA, USA), measuring in the 300–800 nm range with 1 mL of sample in a quartz cuvette (1 cm optical path). Insights into the geometric characteristics of the NPs were obtained by scanning electron microscopy (SEM) using a TESCAN LYRA3 instrument (LYRA) (TESCAN, Brno, Czech Republic), where a 10 μL drop of the sample was deposited on a silicon surface and allowed to dry before imaging. The size distribution of the NPs was analyzed using dynamic light scattering (DLS) measurements using a Malvern Zetasizer Nano ZS instrument (Malvern Panalytical, Malvern, UK), with 2 mL of NP suspension in disposable polystyrene cuvettes at a controlled and constant temperature and a scattering angle of 175° in backscatter mode. The NP concentration and its variation over time were determined via Nanoparticle Tracking Analysis (NTA) using a ViewSizer 3000 by HORIBA (Palaiseau, France), with measurements conducted using a 488 nm laser source. In addition, UV-Vis spectroscopy was employed to characterize the optical absorption properties of the 3D container walls, with measurements taken over the same spectral range (300–800 nm). Finally, a thermal analysis of the container material was performed using thermography with the a U5850 TrueIR Thermal Imager from Keysight (Keysight Technologies, Colorado Springs, CO, USA), capturing temperature distribution profiles through image processing and analysis.

Colorimetric quantification of the TTIs was performed through the analysis of high-quality photographs taken with a Canon EOS 4000D camera (Canon, Tokyo, Japan). Image processing was performed using ImageJ software version 1.54d, developed by the National Institute of Health (Bethesda, MA, USA). To ensure accurate color representation, background light correction was applied, maintaining uniform lighting conditions (600 lux at a 45° angle). The L*, a*, and b* values (lightness, red/green, and yellow/blue coordinates) were extracted for each TTI color. The camera settings were standardized with a white balance of 5500 K, a shutter speed of 1/60 s, an aperture of f/8, and an ISO of 200, ensuring image consistency and preventing exposure problems.

## 3. Results and Discussion

### 3.1. Nanodispersions for TTIs

**Figure 1:**
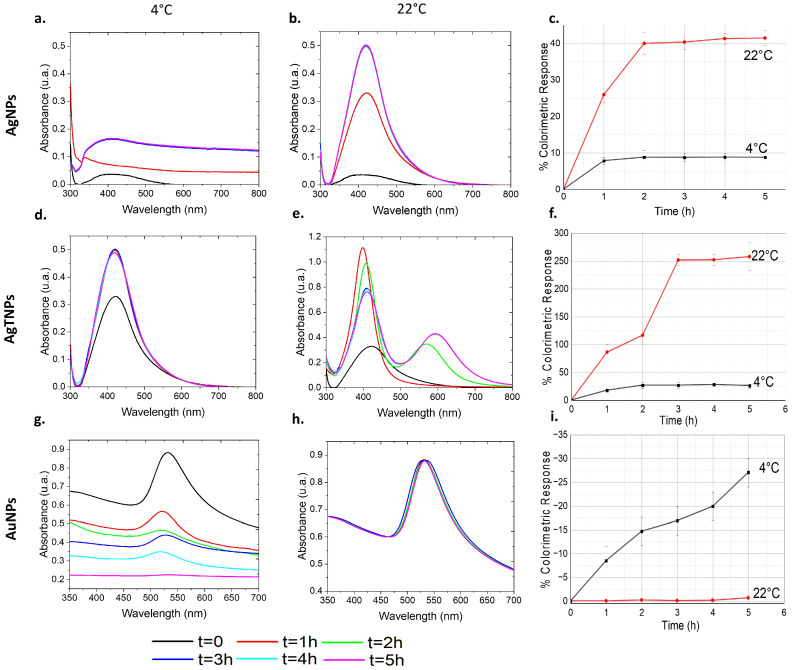
UV-Vis absorption spectra of nanodispersions at 4 °C and 22 °C for AgNPs, AgTNPs, and AuNPs. The first column (**a**,**d**,**g**) shows the nanodispersions at 4 °C, while the second column (**b**,**e**,**h**) displays them at room temperature (22 °C). The third column (**c**,**f**,**i**) compares the estimated colorimetric responses at low temperatures (4 °C) and room temperature (22 °C).

The nanodispersions were prepared and divided into two capped beakers. One beaker was placed on a hotplate to maintain a constant temperature of 22 °C (room temperature), while the other was placed in an ice bath at 4 °C, representing a critical temperature within the cold chain. Throughout this process, temperature monitoring was conducted using a thermocouple to ensure repeatability in the characterization. The beakers were then exposed to various time intervals ranging from 0 to 5 h, allowing for the analysis of the impact of temperature and time on the optical properties of the nanodispersion. At specific time intervals, 1 mL samples of the suspension were carefully extracted from the beakers and transferred into quartz cuvettes and silicon substrates for subsequent characterization. In Figure 1, we present the optical responses of AgNP, AgTNP, and AuNP nanodispersions exposed to temperatures of 4 °C and 22 °C for a duration of 5 h, as determined through UV-Vis absorption spectra.

For AgNPs, as shown in Figure 1a, a slight increase in the absorption peak is observed when exposed to 4 °C, indicating a minimal variation in the optical response. This is in contrast to AgNPs exposed to 22 °C, as depicted in Figure 1b, which display a noticeable increase in peak intensity, with a characteristic absorption peak at approximately 408 nm, which corresponds to the localized surface plasmon resonance (LSPR) of the AgNPs [35]. Notably, in both cases, the absorption peak does not exhibit any wavelength shifts, suggesting a change in color hue rather than a macroscopic color variation. Turning to AgTNPs, Figure 1d illustrates that at 4°C, there is only a slight increase in absorption intensity after the first hour, with subsequent hours showing relatively constant absorption spectra. This behavior differs from the absorption spectra of AgTNPs exposed to 22 °C. Figure 1e reveals that after the first hour, there is an increase in the intensity of the absorption peak at approximately 422 nm. This intensity decreases after 2 h of exposure, while another absorption peak emerges around 572 nm. By the third hour, the first absorption peak decreases again, and the second peak undergoes a shift towards 600 nm before stabilizing. These results macroscopically indicate changes in both color tone and hue. In the case of AuNPs, their temperature-dependent behavior differs from that of AgNPs and AgTNPs. Figure 1g shows absorption spectra centered around 532 nm, which corresponds to the LSPR of the AuNPs [36]. These peaks decrease in intensity at nearly the same wavelength over time when exposed to 4 °C, indicating changes in color tone and opacity. In contrast, Figure 1h illustrates the behavior of AuNPs exposed to 22 °C, which exhibit no appreciable optical changes.

The colorimetric response (%CR) of the nanodispersions is presented in Figure 1c,f,i for AgNPs, AgTNPs, and AuNPs, respectively. It was calculated using the wavelength values from the absorption spectra according to the following equation [37,38]:

(1) $$\%CR=[\left(PB_{0}-PB\right)/PB_{0}]\times 100\%$$

where $PB=A_{blue}/\left(A_{red}-A_{blue}\right)$ is the percentage of blue, $PB_{0}$ is the percentage of blue before exposure, $A_{blue}$ is the absorbance of blue (λ=638 nm), and $A_{red}$ is the absorbance of red (λ=540 nm). When comparing the responses of AgNPs and AgTNPs to different temperatures, similar patterns emerge. At 4 °C, both AgNPs and AgTNPs exhibit initial fluctuations in their colorimetric responses, with AgNPs varying by around 8% and AgTNPs by approximately 18%. However, these variations stabilize over time, with AgNPs maintaining a consistent %CR within a 1% range and AgTNPs remaining steady after 2 h. In contrast, exposure to 22 °C yields more pronounced changes. AgNPs show an increase in %CR, reaching 25% after just 1 h, followed by a steady climb to 40% after 2 h. Beyond this, the increase slows noticeably, indicating a saturation effect. Similarly, AgTNPs exposed to 22 °C display an even more pronounced response, with an 86% increase in %CR after 1 h, escalating to 116% after 2 h and a staggering 252% after 3 h. This steep rise illustrates the substantial shift in color hue experienced by AgTNPs under warmer conditions. Notably, AuNPs respond differently to temperature variations. At 22 °C, their %CR remains relatively stable, with minimal fluctuations of less than 1%. Conversely, exposure to 4 °C leads to a progressive decrease in %CR over time, signaling a notable alteration in color. By the end of the 5 h period, the %CR for AuNPs reaches up to 27%, highlighting the sensitivity of AuNPs to colder temperatures.

### 3.2. Operation Strategies of the Active Material for TTIs

**Figure 2:**
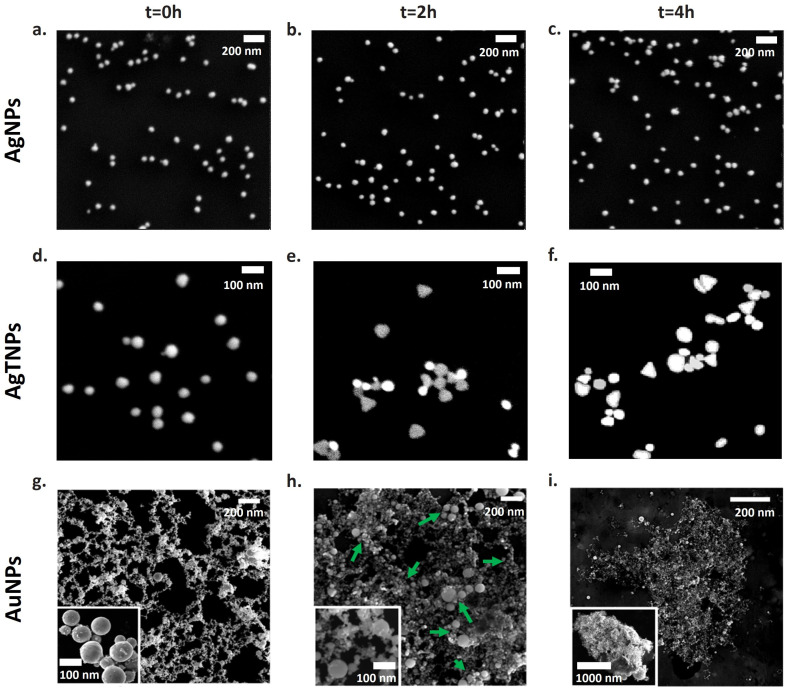
Time-dependent morphological changes of the nanodispersions in SEM images. In the first column, they are listed for the initial time (t = 0 h), in the second column for an intermediate time (t = 2 h), and in the third column for a time of t = 4 h for AgNPs (**a**–**c**) and AgTNPs (**d**–**f**) at 22 °C and for AuNPs (**g**–**i**) at 4 °C. The green arrows in (**g**) indicate points of NPs agglomeration.

To investigate the strategies by which nanodispersions alter their colorimetric response at temperatures of 22 °C for AgNPs and AgTNPs and 4 °C for AuNPs, SEM images were processed and analyzed at 0, 2, and 4 h of exposure. For each time point, three random images per sample were analyzed, with particle counts ranging from 20 to 60 NPs. These images provided insights into the size and morphology of the nanoparticles. The SEM images in Figure 2a–c depict AgNPs with a spherical appearance at all exposure times, with NPs having an average diameter of 32.8 ± 5 nm and a polydispersity index (PDI) of 0.49, as determined by DLS characterization. These results suggest that, for AgNPs, the colorimetric response mechanism is not based on changes in shape and/or size.

**Figure 3:**
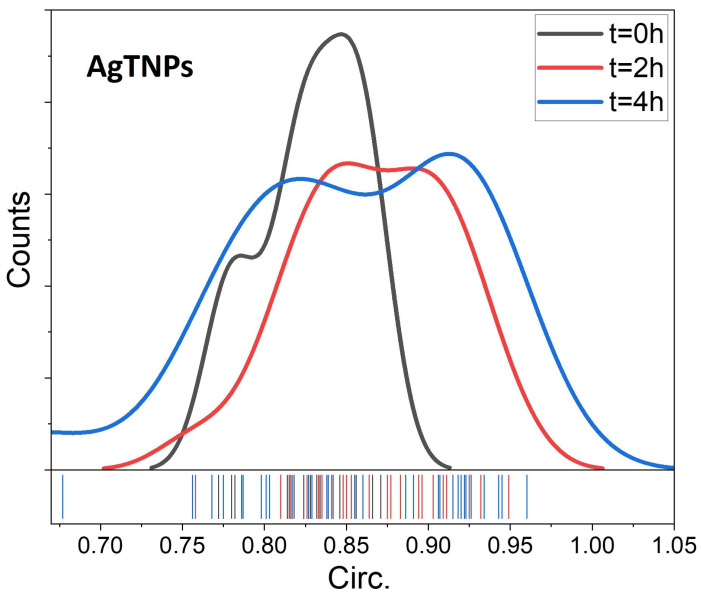
Changes in the circularity distribution of the AgTNPs evolving after 0, 2, and 4 h at an ambient temperature of 22 °C.

On the other hand, analysis of the SEM images in Figure 2d–f reveals changes in the shape and percentage of AgTNPs. Initially, AgTNPs exhibit a spherical shape with a diameter of 43.3 ± 10 nm and a polydispersity index of 0.32. However, at t = 2 h and t = 4 h, NPs with elliptical and triangular shapes, in addition to spherical ones, are observed. To quantify the shape changes induced in AgTNPs by exposure to temperature, circularity was used as a shape descriptor. Circularity is defined as follows [39]:

(2) Circ.=4πA/p

where *A* is the area and *p* is the perimeter. A circularity value of 1 indicates a perfect circle, while values approaching 0 suggest an increasingly elongated shape. By quantifying circularity, it is possible assess the deviation of NP shapes from a perfect circular form, revealing any significant changes in their morphology over time [40]. In Figure 3, the circularity distributions of AgTNPs exposed at 22 °C exhibit notable variations in the curves as time advances. These distributions are centered within a range between 0.81 and 0.92, with an increasing widening of values over time, indicating a greater dispersion for this descriptor.

The circularity distributions of AgTNPs reveal morphological changes in a portion of the AgTNPs over time when exposed to a temperature of 22 °C. This suggests that altering their shape is the mechanism behind the change in the colorimetric response of the nanodispersion. The presence of elliptical and triangular NPs indicates that temperature-induced transformations in the AgTNPs lead to a diverse range of particle shapes, subsequently affecting the optical properties of the nanodispersion. The mechanism presented in AgTNPs is rooted in the variation of the geometry of a certain percentage of NPs, leading to heterogeneity in the LSPR of NPs, which is defined as a multispectral LSPR, and subsequently affecting their overall colorimetric response. The relationship between the initial LSPR peak position and temperature sensing relies on the geometry of NPs within the nanodispersion [41]. We attribute the geometric shift of a portion of the NPs to the influence of temperature in the employed synthesis method (seed-based) [22]. For these AgTNPs, AgNP seeds were utilized, acting as nuclei onto which additional Ag was deposited, enabling controlled growth and formation of new nanoparticles with shapes strongly influenced by exposure time and temperature [42]. To quantify the percentage of AgTNPs undergoing changes in their initial circularity due to exposure to a temperature of 22 °C for 5 h, we conducted a deconvolution of the absorption spectra of the AgTNPs. This deconvolution assigned absorption peaks to NPs with specific ranges of circularity within the dispersion. Over time, the initial percentage, originally at 100% and associated with the initial morphology, decreased to 65.1% after 2 h of exposure and further to 56.6% after 5 h. As this initial percentage decreased, a second peak emerged after 2 h of exposure, representing 34.9% and eventually reaching a maximum of 43.5%. This second peak corresponds to NPs with a morphology distinct from that of the initial NPs.

For AuNPs (Figure 2g–i), agglomeration processes were observed at low temperatures. At the initial time point (t = 0 h), spherical NPs with a size distribution centered around 126.2 ± 5.7 nm and a PDI of 0.151 (determined through DLS) were identified. As the exposure time at 4 °C increased to 2 and 4 h, the SEM images clearly displayed a progressive increase in the agglomeration of AuNPs. This time-dependent agglomerate formation at low temperatures serves as the underlying mechanism responsible for the observed changes in the colorimetric response of these nanoparticles. Authors have reported such agglomeration processes for AuNPs at temperatures below 0 °C (down to −196 °C), referring to them as cryoagglomeration processes [43]. These processes are attributed to the interplay between attractive Van der Waals forces among the NPs in suspension and the repulsive electrostatic forces generated by the protective agents used during the synthesis of AuNPs [44]. However, in the case of the AuNPs physically synthesized in this study, no additional material coatings were generated, meaning that the interaction between the NPs is governed solely by attractive Van der Waals forces, promoting agglomeration, even at temperatures of 4 °C [45]. At low temperatures, the average interparticle distances between NPs decrease, and the electrostatic interactions among the NPs intensify, leading to the agglomeration. Consequently, a plasmonic coupling effect occurs, resulting in a collective colorimetric response [46].

**Figure 4:**
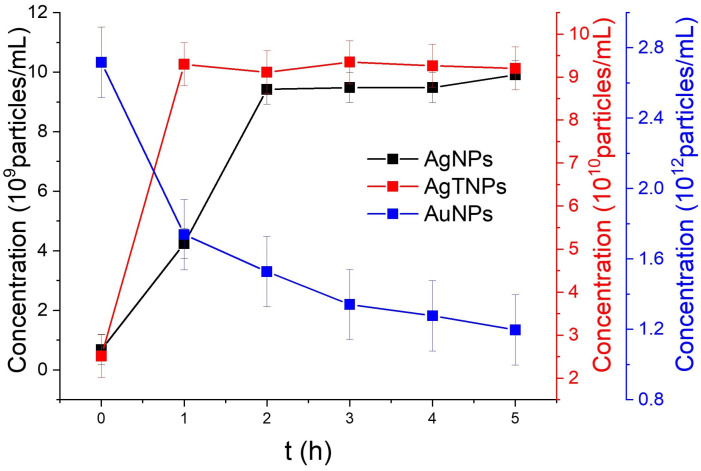
Concentration of AgNPs and AgTNPs exposed to 22 °C and AuNPs exposed to 4 °C for different periods of time.

Concentration measurements using NTA were conducted for all three nanodispersions. The NP concentration was assessed at specific temperatures that induced significant colorimetric changes: 22 °C for AgNPs and 4 °C for AuNPs, over a 5-h period. In Figure 4, the concentration of AgNPs demonstrates an increase during the initial 3 h of exposure to the designated temperature, rising from 6.82 $\times \;10^{8}$ to 9.42 $\times \;10^{9}$ NPs/mL. This implies that the chemical synthesis process used for these AgNPs benefits from thermal enhancement, leading to greater NP production over time. Subsequently, over the following two hours, there is a slight increase in concentration, from 9.47 $\times \;10^{8}$ to 9.90 $\times \;10^{9}$ NPs/mL. This observed rise in NP concentration underscores the dynamic nature of the nanodispersion system when subjected to temperature fluctuations. As the synthesis process continues within the dispersion, it generates a greater number of NPs, thereby altering the colorimetric properties attributed to plasmonic coupling. This operational mechanism relies on the temperature-dependent nature of the synthesis method (chemical reduction), where metallic ions are converted into NPs through the reduction of chemical precursors. Temperature increases the rate of the reduction reaction due to increased kinetic energy of the involved molecules [47].

For AgTNPs, there is an initial increase in concentration during the first hour, after which it stabilizes at around 9.30 $\times \;10^{9}$ NPs/mL, indicating synthesis process stability at room temperature. This finding suggests that variations in the colorimetric response of AgTNPs may not primarily result from the generation of new NPs through a chemical synthesis process. Finally, for AuNPs, the concentration analysis reveals a decrease in concentration values from 2.7 $\times \;10^{12}$ to 1.19 $\times \;10^{12}$ NPs/mL. This observation points to possible agglomeration effects occurring in the nanodispersion, which consequently reduce the count of individual NPs within the suspension.

### 3.3. Functional Time–Temperature Indicators (TTIs)

**Figure 5:**
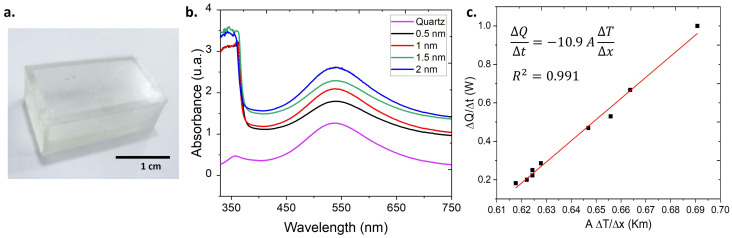
Containers designed by 3D printing using stereolithography (SLA): (**a**) photograph of the printed container; (**b**) absorption spectrum of AuNPs in the container; (**c**) estimation of the thermal conductivity of the container material.

Based on the definition of TTI in this study (a system comprising a nanodispersion and a container), containers designed to store the synthesized and characterized nanodispersions were produced (Figure 5a). For the optical characterization of the container material, cuvettes with dimensions and geometry identical to those of the commercial quartz cuvettes (side length of 12.5 mm) used in the UV-Vis spectrophotometer were also 3D-printed. The wall thickness of the printed cuvettes ranged from 0.5 to 2 mm, ensuring a consistent optical path length for measurements. The absorption spectra in Figure 5b display the absorption of AuNPs contained in both a commercial quartz cuvettes and the printed cuvettes. UV-Vis spectra indicate that the plant-based resin cuvette does not alter the position of the characteristic absorption peak of the AuNPs (only varying in intensity with respect to the thickness of the walls). This suggests that, optically, the material used in the fabrication of the containers is suitable. The calculated values from the thermographic measurements are presented in Figure 5c. Based on the obtained results, a thermal conductivity of κ=10.9±0.5 W/Km was determined for the plant-based resin used as the container material [48]. In the literature, thermal conductivity values for AgNPs and AuNPs suspended in water are reported in the ranges of 0.05 to 2 W/m·K and 0.1 to 5 W/m·K, respectively [49,50]. Comparatively, the value of κ for the container material is higher than that of the nanodispersions, allowing for appropriate thermal transfer from the environment to the NPs.

**Figure 6:**
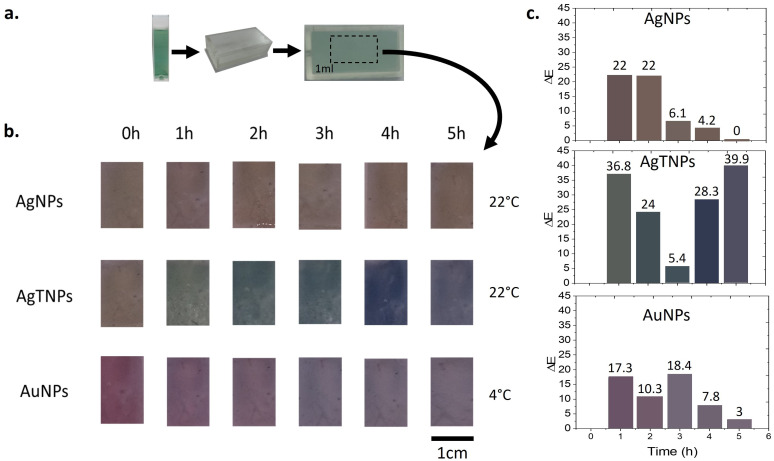
Photographs of the functional TTIs (nanodispersion in the container) for optical response characterization. (**a**) Nanodispersion, container, and TTI for characterization; (**b**) photographs of the functional TTIs for color identification; (**c**) total color difference (ΔE) for the functional TTIs.

To produce functional TTIs, nanodispersions were introduced into custom-designed containers, as illustrated in Figure 6a. Upon filling, the containers were sealed with acrylic resin, effectively encapsulating the nanodispersions. The colorimetric quantification of the TTIs was conducted by analyzing photographs of the colorimetric responses at the reaction temperatures for AgNPs (22 °C), AgTNPs (22 °C), and AuNPs (4 °C), as shown in Figure 6b. For each TTI, the average color was determined over a time range of 0 to 5 h. Color information from the photographs was extracted and quantified, allowing for precise measurement of color changes using the total color difference (ΔE). The total color difference was calculated using the following equation [51]:

(3) $$\Delta E=\sqrt{{\left(\Delta L^{*}\right)}^{2}+{\left(\Delta a^{*}\right)}^{2}+{\left(\Delta b^{*}\right)}^{2}},$$

where L* is the lightness, a* is the red/green coordinate, and b* is the yellow/blue coordinate. By calculating differences in these components, ΔE provides a comprehensive measure of the overall color change between samples. According to the ISO 12647 standard [52,53,54], it is possible to assign qualitative judgments to the colorimetric changes experienced by a TTI based on the range of ΔE values. For ΔE≥5, the colorimetric changes would be noticeable to any average observer.

In Figure 6c, the results of the total color difference for TTIs are presented. The bars in the graphs show the color of the TTIs exposed at temperatures of 22 °C for AgNPs and AgTNPs and 4 °C for AuNPs. For TTIs based on AgNPs, a color difference of 22 is observed during the 1st hour, and this value remains constant for another hour. However, from the 2nd to the 3rd hour, the color difference decreases to 6.2. Continuing from the 3rd to the 4th hour, the color difference further decreases to 4.3. Finally, from the 4th to the 5th hour, no color difference is observed. This indicates that the operational time range for these TTIs is limited to 4 h. When comparing AgTNP TTIs to AgNP TTIs, the total color difference values are distinguishable by an observer in all cases. For the AgtNPs, in the 1st hour, the color difference reaches 36.8, followed by a decrease to 24 between the 1st and 2nd hours. It then reaches a minimum value of 5.5 between the 2nd and 3rd hours. Subsequently, from the 3rd to the 4th hour, the color difference increases to 28.3, reaching a maximum value of 39.9 between the 4th and 5th hours. This trend is attributed to variations not only in color tone but also in hue for these TTIs. As for the TTIs based on AuNPs, during the 1st hour, the color difference is 17.4, followed by a value of 10.3 between the 1st and 2nd hours. In the subsequent time intervals, the difference increases again to 18.4. From the 3rd to the 4th hour, the color difference decreases to 7.8, and finally, from the 4th to the 5th hour, it reaches a minimum value of 3.

Table 1 summarizes the technical specifications of the operational characteristics of the functionals TTIs. The colorimetric response of these TTIs implies an irreversible color change over time when exposed to temperatures outside the optimal cold chain range (temperatures > 4 °C) for both compositional TTIs. In contrast, for TTIs based on AuNPs, the irreversible color change occurs when exposed to low temperatures (temperatures < 4 °C); therefore, they could be applied to perishable products that exhibit chilling injury damage [55]. The operational time for the TTIs is up to 4 h for those based on AgNPs and AuNPs, while the TTIs incorporating AgTNPs offer an extended operational time of up to 5 h. To ensure the effectiveness of the TTIs, it is crucial to keep them in an inactive state when not in use to prevent unintended activation, as indicated in the table.

**Figure 7:**
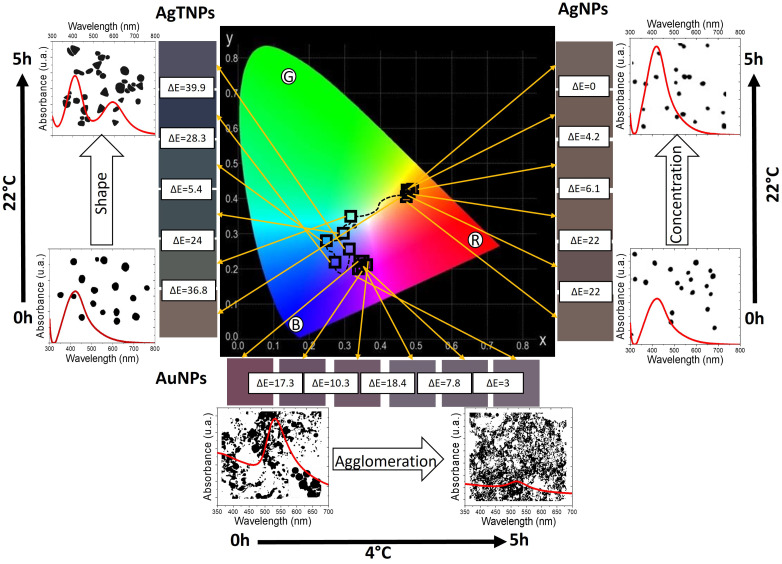
Cielab color space, total color difference, activation mechanisms, optical response, operation time, and activation temperature for functional TTIs based on AgTNPs, AgNPs, and AuNPs.

In Figure 7, we present a compound chromaticity graph that correlates the characteristics of the TTIs based on AgTNPs, AgNPs, and AuNPs engineered in this work. This graph facilitates an interdisciplinary understanding of the presented nanoscopic and macroscopic results. The graph, moving from the center outward, displays the colorimetric trajectory of the TTIs over time on a CIE chromaticity diagram, followed by the total color difference of each TTI, indicating its respective activation mechanism and optical response and, finally, the operational time and activation temperature. TTIs fabricated with AgTNPs (on the left) exhibit a colorimetric response starting in the greenish–yellow region, transitioning through the blue–green region and reaching the purplish–blue region. The mechanism for AgTNPs is based on changes in the shape of the nanoparticles at a temperature of 22 °C, resulting in colorimetric changes (tone and hue) that are easily noticeable to an observer, with maximum total color differences reaching up to 39.9 within a 5 h operational range. For AgNP-based TTIs (on the right), the color trajectory remains within the greenish–yellow region. The response mechanism of AgNPs operates through changes in nanoparticle concentration, resulting in colorimetric variations in tone over a 4 h operational period at 22 °C. These results demonstrate the correlation between the colorimetric change mechanism and the method of Ag nanoparticle synthesis. TTIs incorporating AuNPs (at the bottom) show color changes primarily in the reddish–purple region. For AuNPs, it was found that their colorimetric response occurred at low temperatures (4 °C) due to nanoparticle agglomeration processes, resulting in noticeable changes in total color difference of up to 17.4 within the first 4 h of operation.

## 4. Conclusions

In this study, we engineered functional time–temperature indicators (TTIs) using AgNPs, AgTNPs, and AuNPs. These functional TTIs serve as tags to detect breaks in the cold chain by monitoring temperature changes. We explored three distinct operational strategies to understand how these suspended NPs respond to temperature exposure over time. For AgNPs and AgTNPs, the temperature was set at 22 °C, while AuNPs were examined at 4 °C, each over a 5 h period. Our findings highlight the specific strategies adopted by each NP type. AgNPs rely on an increase in nanoparticle concentration within the nanodispersion, driven by a temperature-dependent synthesis method involving chemical reduction. At 22 °C, the accelerated reduction reaction rate enhances this concentration effect. For AgTNPs, the mechanism involves geometric alterations in a fraction of the NPs, creating heterogeneity in their localized surface plasmon resonance (LSPR). This process uses AgNP seeds as nuclei, with exposure time and temperature significantly influencing NP shapes within the TTI. AuNPs, on the other hand, respond through agglomeration processes at lower temperatures (<4 °C), where reduced interparticle distances heighten electrostatic interactions, promoting NP agglomeration and resulting in a collective colorimetric response due to plasmonic coupling. The 3D-printed containers used in this study, made from plant-based resin, are well-suited for housing the nanodispersions, exhibiting appropriate transparency and thermal conductivity. The developed functional TTIs, featuring a colorimetric indicator, demonstrate significant potential as indispensable and easily interpretable tools for both consumers and food producers. Among the tested functional TTIs, those based on AgTNPs exhibit the most distinguishable colorimetric changes at 22 °C, resulting in a total color difference ΔE of 39.9 (easily noticeable to an average observer ΔE≥ 5, ISO 12647). By securely affixing these colorimetric indicators to food packaging, continuous and real-time monitoring of the cold chain is enabled, ensuring precise monitoring of cold chain maintenance of optimal conditions for food safety and quality.

## Figures and Tables

**Table 1:** Technical specifications for manufactured functional TTIs.

	AgNPs	AgTNPs	AuNPs
Indication Type	Visual, irreversible greenish–yellow tone change in activation window	Visual, irreversible color tone and hue change in activation window, from greenish–yellow, transitioning through blue–green to purplish–blue.	Visual, irreversible reddish–purple tone change in activation window
Activation Temperature	>22 °C	>22 °C	<4 °C
Recording Time	4 h	5 h	4 h
Mounting Method	Adhesive label on the packaging		
Storage Conditions	Store in a cool, dark environment below 4 °C		Store at room temperature in the dark
Dimensions	2.1 cm × 1.1 cm × 0.5 cm		

## Data Availability

The original contributions presented in this study are included in the article. Further inquiries can be directed to the corresponding author.

## Abbreviations

The following abbreviations are used in this manuscript:

AgNPs	Silver Nanoparticles
AgTNPs	Silver Triangular Nanoparticles
AuNPs	Gold Nanoparticles
DLS	Dynamic Light Scattering
FAO	Food and Agriculture Organization of the United Nations
LSPRs	Localized Surface Plasmon Resonances
MNPs	Metal Nanoparticles
NPs	Nanoparticles
NTA	Nanoparticle Tracking Analysis
SDGs	Sustainable Development Goals
SEM	Scanning Electron Microscopy
TTIs	Time–Temperature Indicators
UV-Vis	UV-Visible Absorbance Spectroscopy
